# Implementation of a Spatially-Variant and Tissue-Dependent Positron Range Correction for PET/CT Imaging

**DOI:** 10.3389/fphys.2022.818463

**Published:** 2022-03-08

**Authors:** Hunor Kertész, Thomas Beyer, Vladimir Panin, Walter Jentzen, Jacobo Cal-Gonzalez, Alexander Berger, Laszlo Papp, Peter L. Kench, Deepak Bharkhada, Jorge Cabello, Maurizio Conti, Ivo Rausch

**Affiliations:** ^1^QIMP Team, Center for Medical Physics and Biomedical Engineering, Medical University of Vienna, Vienna, Austria; ^2^Siemens Medical Solutions USA, Inc., Knoxville, TN, United States; ^3^Clinic for Nuclear Medicine, University Hospital Essen, Essen, Germany; ^4^Ion Beam Applications, Quirónsalud Proton Therapy Center, Madrid, Spain; ^5^Discipline of Medical Imaging Science and Brain and Mind Centre, Faculty of Medicine and Health, The University of Sydney, Sydney, NSW, Australia

**Keywords:** positron emission tomography, image reconstruction, positron range correction, PET quantification, PRC

## Abstract

**Aim:**

To develop and evaluate a new approach for spatially variant and tissue-dependent positron range (PR) correction (PRC) during the iterative PET image reconstruction.

**Materials and Methods:**

The PR distributions of three radionuclides (^18^F, ^68^Ga, and ^124^I) were simulated using the GATE (GEANT4) framework in different material compositions (lung, water, and bone). For every radionuclide, the uniform PR kernel was created by mapping the simulated 3D PR point cloud to a 3D matrix with its size defined by the maximum PR in lung (^18^F) or water (^68^Ga and ^124^I) and the PET voxel size. The spatially variant kernels were composed from the uniform PR kernels by analyzing the material composition of the surrounding medium for each voxel before implementation as tissue-dependent, point-spread functions into the iterative image reconstruction. The proposed PRC method was evaluated using the NEMA image quality phantom (^18^F, ^68^Ga, and ^124^I); two unique PR phantoms were scanned and evaluated following OSEM reconstruction with and without PRC using different metrics, such as contrast recovery, contrast-to-noise ratio, image noise and the resolution evaluated in terms of full width at half maximum (FWHM).

**Results:**

The effect of PRC on ^18^F-imaging was negligible. In contrast, PRC improved image contrast for the 10-mm sphere of the NEMA image quality phantom filled with ^68^Ga and ^124^I by 33 and 24%, respectively. While the effect of PRC was less noticeable for the larger spheres, contrast recovery still improved by 5%. The spatial resolution was improved by 26% for ^124^I (FWHM of 4.9 vs. 3.7 mm).

**Conclusion:**

For high energy positron-emitting radionuclides, the proposed PRC method helped recover image contrast with reduced noise levels and with improved spatial resolution. As such, the PRC approach proposed here can help improve the quality of PET data in clinical practice and research.

## Introduction

Positron emission tomography (PET) is a widely used non-invasive imaging method to visualize and quantify functional and metabolic processes for the diagnosis, staging, and follow-up of disease (Muehllehner and Karp, 2006). In PET, a positron-emitting radionuclide is attached to a biomolecule, aka tracer, which is administered to the subject, and the three-dimensional (3D) tracer distribution is then reconstructed from measurements of the emitted radiation (Ziegler, 2005).

The positron emitted from the radionuclide travels a finite distance while interacting with electrons in the surrounding tissues until it annihilates, thus producing two co-linear gamma photons that can be detected by the surrounding PET detectors. Thus, the measured tracer distribution reflects the positron annihilation point distribution rather than the initial emission point, which represents the true location of the tracer.

The distance traveled by the positrons from the point of emission to the annihilation point is referred to as positron range (PR) and depends on the energy of the emitted positron and the electron density of the surrounding medium. In the case of ^18^F, which is the most commonly used radionuclide in PET, the positron emission energies are relatively low (maximum positron energy: Emax = 0.63 MeV, mean positron energy: Emean = 0.25 MeV), and the mean PR (rmean) in water is only 0.6 mm (Conti and Eriksson, 2016). This does not induce considerable differences between the measured and true tracer distribution (Alessio and MacDonald, 2008), given the spatial resolution of state-of-the-art PET systems ranges from 2 to 4 mm (Delso et al., 2011; Rausch et al., 2015, 2019; Grant et al., 2016; Cal-Gonzalez et al., 2018a).

However, when radionuclides with high positron emission energies and complex decay schemes (e.g., ^68^Ga, Emax = 1.9 MeV, rmean = 2.9 mm), ^124^I with two positron emissions resulting in two positron ranges (Emax1 = 1.54 MeV, rmean1 = 2.8 mm, Emax2 = 2.14 MeV, rmean2 = 4.4 mm; Jødal et al., 2012, 2014; Conti and Eriksson, 2016; Emond et al., 2019) are used, notable deterioration of the perceived spatial resolution resulting in image blurring and loss of image contrast (Cho et al., 1975; Michael et al., 1975; Hoffman et al., 1976). ^68^Ga labeled tracers showed benefits in diagnostics of prostate cancer patients (Singh et al., 2018; Hoffmann et al., 2019), likewise ^124^I has been used with patients diagnosed with thyroid cancer (Herzog et al., 2010; Freudenberg et al., 2011).

To overcome the limitations induced by the PR effects, several PR correction (PRC) methods have been proposed (Derenzo, 1986; Bai et al., 2003; Rahmim et al., 2008; Fu and Qi, 2010; Kraus et al., 2012; Cal-González et al., 2015; Moreau et al., 2015; Bertolli et al., 2016), most of which assume a spatially uniform Gaussian PR distribution (Derenzo, 1986). However, fewer, yet more realistic approaches, consider a heterogeneous tissue-dependent distribution following an isotropic Gaussian distribution within a specific tissue (Bai et al., 2003; Alessio and MacDonald, 2008; Cal-González et al., 2015). All of these studies assume that the positron range distribution can be modeled from Monte Carlo (MC) simulations or calculated analytically. MC simulation frameworks help model uniform PR distributions within a given material as well as spatially variant PR distributions.

However, modeling the spatially variant kernels for every voxel is computationally intensive and not suitable for clinical use. Analytically derived models of PR distributions are based on fitting the proposed PR function to the maximum PR (Cal-González et al., 2013). The spatial variation can be achieved by scaling the analytically calculated PR distribution by the mean density between the emission and annihilation points. This approximation is suitable for the clinical workflow, however, is less accurate, especially when the relationship between electronic density and positron range is not linear (Cal-González et al., 2015). PR kernels derived from these modeled PR distributions can be applied to the PET images either as an image deconvolution or as an additional point-spread function of the current image update during the image reconstruction process, with the latter having the disadvantage of slowing down the image reconstruction process (Bertolli et al., 2016; Cal-Gonzalez et al., 2018a).

Despite these methodological advances, PRC is not yet adopted routinely. This need is further amplified by the recent application of higher-energy positron emitters in theranostics for example ^68^Ga DOTATATE (Ballinger, 2018; Harvey Turner, 2018; Könik et al., 2021).

This study aimed to develop a fast and efficient method to estimate spatially variant and tissue-dependent positron range distributions. The resulting PRC based on these distributions should be implemented within a vendor-based image reconstruction software for ease-of-use applications in clinical PET/CT.

## Materials and Methods

### Positron Range Distribution Simulations

Positron range distributions were simulated in GATE 9.0 (GEANT4 10.06.p02; Jan et al., 2004, 2011). Three different radionuclides (^18^F, ^68^Ga, and ^124^I) were simulated in combination with three different material compositions of the phantom: lung (mass density 0.26 g/cm^3^), water (1.00 g/cm^3^), and bone (1.92 g/cm^3^), as defined in the GATE material database. The simulation geometry consisted of a point source (diameter of 10 nm) centered in a uniform spherical phantom (*d* = 60 cm). The simulation setup was centered in the FOV. The low energy physics list “empenelope” was used with an initial activity of 10 MBq and 20 million simulated annihilation events. The emission coordinates (*x*_i_, *y*_i_, *z*_i_), which represent the center of the uniform spherical phantom and the annihilation coordinates (*x*_f_, *y*_f_, *z*_f_) of the positrons were recorded and used for subsequent analyses. The distance traveled by the positrons was calculated as:


(1) $$r=\sqrt{{\left(x_{f}-x_{i}\right)}^{2}+{\left(y_{f}-y_{i}\right)}^{2}+{\left(z_{f}-z_{i}\right)}^{2}\;}$$

For all radionuclide and material combinations considered, the mean (*r*_mean_) was calculated and compared to previous studies.

### Positron Range Kernel Calculation

Positron range kernels in uniform materials were obtained from mapping the positron annihilation points to a 3D grid and normalizing the kernels to the area. The dimensions of the 3D grid were chosen according to the maximum positron range for the given radionuclide in the lung medium for ^18^F and the maximum range in water for ^68^Ga, and ^124^I and the voxel size of the PET system.

### Generating Spatially Variant Kernels

**Figure 1:**
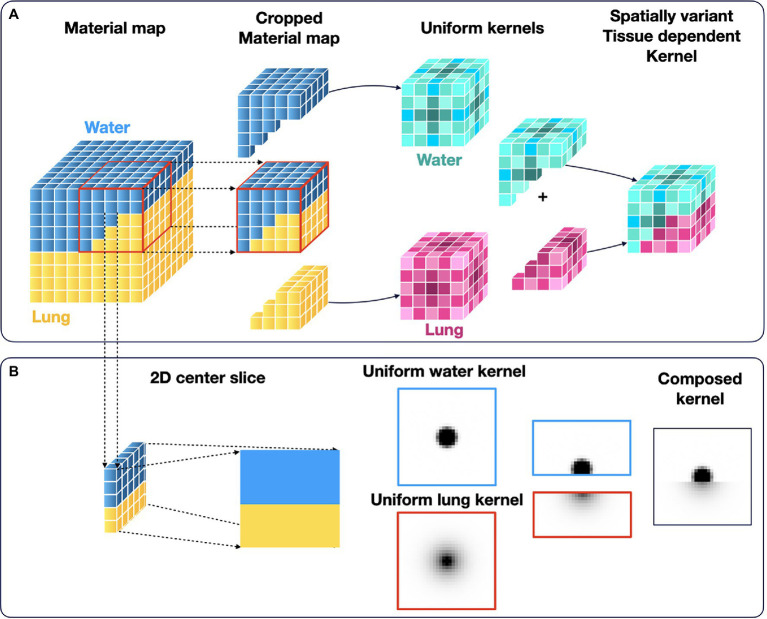
Calculation ossf the spatially variant and tissue-dependent positron range kernel. First, the local material composition was obtained from the material map (CT used for attenuation correction). Then, the tissue-specific kernels were combined accordingly to a spatially variant, tissue-dependent kernel. **(A)** Schematics of the method in 3D, and **(B)** Sample of central 2D slice of the combined kernel from the uniform ^124^I water and lung kernels.

To limit computational demand, spatially variant kernels were approximated by a simple material-dependent combination of the uniform 3D kernels (Figure 1A). The local variations in tissue density were derived from the generated attenuation correction map. The image and voxel size of the AC maps and the PET images is the same. For each voxel, the surrounding material composition was determined from the attenuation map in an area with a size similar to the radionuclide-dependent kernel size. Then, a voxel-specific new kernel was composed by combining the respective homogeneous kernel parts depending on the corresponding voxels of the uniform kernels. Finally, the voxel-specific kernel was normalized to the area. Figure 1B shows an example for combining the uniform ^124^I kernels.

**Figure 2:**
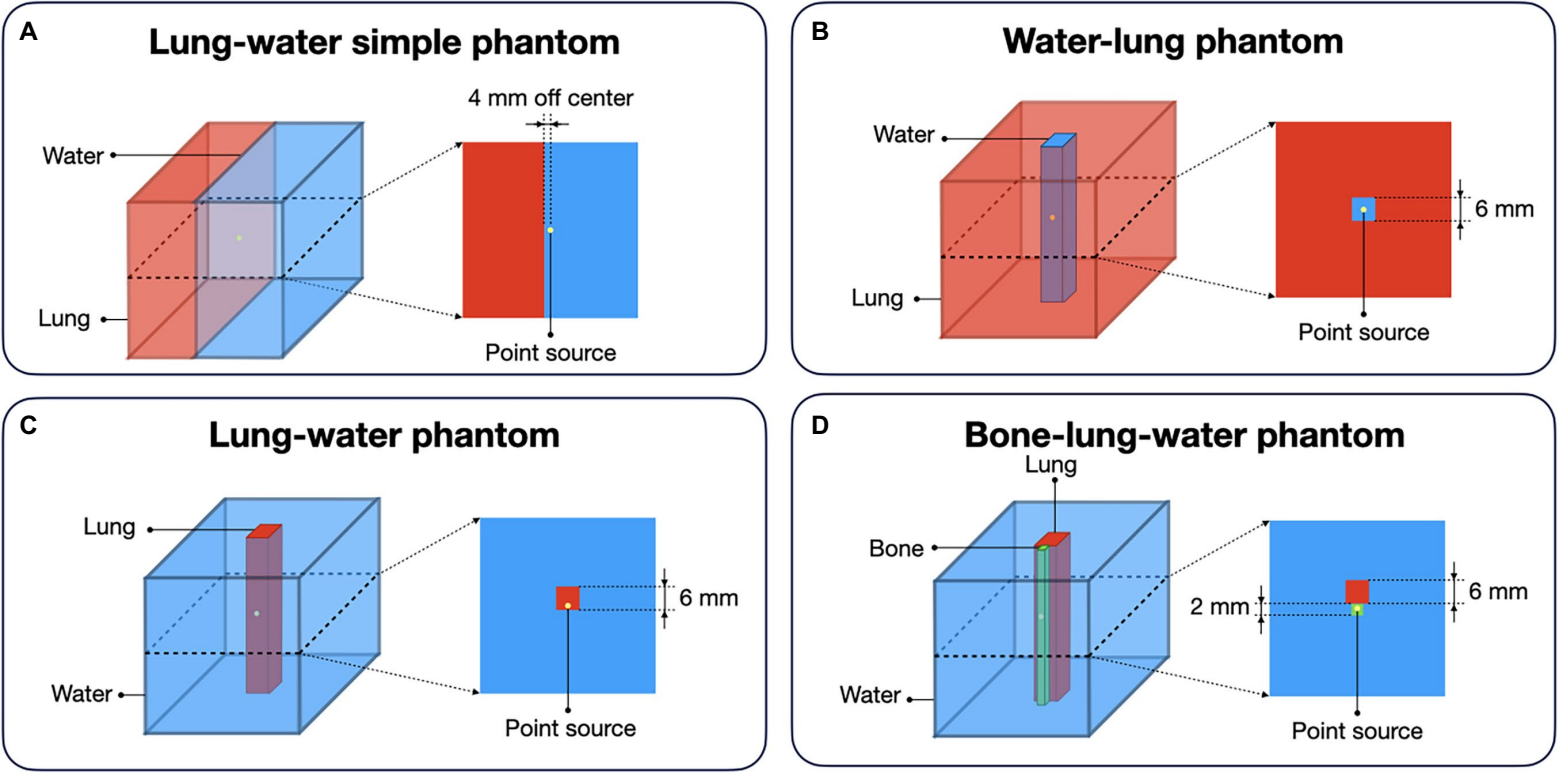
Different digital phantom compositions for the evaluation of the proposed method for the calculation of the spatially variant positron range kernels. For every phantom, the point source was placed in the center of the 3D volume: **(A)** Lung–water simple phantom: simulation of a border between lung and water with the source being covered in water; **(B)** Water–lung phantom: simulation of the shine through effect with placing a water cuboid in lung medium; **(C)** Lung–water phantom: cuboid made of lung placed in water medium with the source set offset; **(D)** Bone–lung–water phantom: point source placed in bone material next to a lung material in water medium.

The positron range distribution was simulated for four tissue composition scenarios and compared with the proposed simple kernel composition method. An ^124^I point source (*d* = 1 nm, initial activity of 10 MBq) was placed in the center of a 10 × 10 × 10 cm^3^ box phantom composed of different materials (Figure 2). (a) Water–lung simple phantom with a border between lung and water was simulated with the source covered by water and the neighboring lung material being 4 mm off-center to simulate a simple border between a high- and low-density material (Figure 2A). (b) Water–lung phantom, which corresponds to a 0.6 × 0.6 × 10 cm water cuboid centered in lung tissue, to simulate a lung lesion (Figure 2B). Next, we use a Lung–water phantom: a 0.6 × 0.6 × 10 cm lung cuboid was placed 4 mm off-center within water representing a lesion in the airways (Figure 2C). And finally, a Bone–lung–water phantom was simulated by means of a 0.2 × 0.2 × 10 cm cuboid of bone covering the point source was centered within the water-filled phantom, and, additionally, a 0.6 × 0.6 × 10 cm cuboid composed of lung was positioned next to the bone insert to simulate a more complex scenario of lung, soft tissue and rib bone (Figure 2D). For every simulation, 20 million annihilation events were collected.

### Implementation of the PRC into Image Reconstruction

The implementation of the PRC into the OSEM algorithm did follow the description of Reader et al. (2002). The simplified OSEM algorithm implemented in the vendor tools was written as follows (background events, such as randoms and scatter were omitted):


(2) $$f_{j}^{n+1}=\frac{f_{j}^{n}}{\sum_{i\prime \in S^{n}}a_{i\prime j}}\sum_{i\in S^{n}}a_{ij}\frac{m_{i}}{\sum_{k}a_{ik}f_{k}^{n}}$$

where *f_j_^n + 1^* is the next image estimate of voxel *j* based on the current image estimate *f_j_^n^*. *m_i_* is the measured projection data and *a_ij_* is describing the probability of the emission from voxel *j* will be detected along the line of response (LOR) *i*. Only a subset *S^n^* of the data was used in each update (Alessio and Kinahan, 2006).

The matrix of probabilities *A = (a_ij_)_IxJ_* can be factorized (Reader et al., 2002):


(3) A=WXH

where the matrix *H = (h_j’j_)_JxJ_* includes the finite resolution effects, in our study the PR effects. Matrix *X = (x_ij_)_IxJ_* is the matrix describing the intersection lengths as above, and *W = (w_ii_)_IxI_* takes into account geometric sensitivity variations and photon attenuation. With this, Eq. (2) can be rewritten as:


(4) $$f_{j}^{n+1}=\frac{f_{j}^{n}}{\sum_{b}h_{bj}\sum_{i\in S^{n}}w_{ii}x_{ib}}\sum_{b}h_{bj}\sum_{i\in S^{n}}x_{ib}\frac{m_{i}}{\sum_{p}x_{ip}\sum_{v}h_{pv}f_{v}^{n}}$$

For simplification, the ordered subsets expectation maximization (OSEM) with the added positron range modeling described above is called positron range correction (PRC) in this study. All other steps within the iterative reconstruction were performed as implemented in the original reconstruction by the vendor.

The proposed PRC method was implemented into a modified version of the Siemens e7tools image reconstruction software (Siemens Medical Solutions USA, Inc., Knoxville, TN, United States), which allows to pass an image guess as input and returns the back-projected correction image. The core implementation of the method was performed using an in-house pipeline using MATLAB R2019a (Mathworks Inc., United States). The practical implementation of the PRC methods was done by applying the transpose of the PR convolution operator to the correction image, correcting the initial image, and then applying the PR convolution operator to the corrected image before passing it again to the modified e7tools. During the image reconstruction, the spatially variant kernels were calculated for every voxel as explained in the section above. The calculation of the spatially variant kernels was programmed using C++ (Qt Framework) as a parallel approach using multiple CPUs.

### Experimental Evaluation

The proposed PRC method was validated using three different phantom measurements (see below for details). All phantom acquisitions were performed for a single bed position using a Siemens Biograph mCT PET/CT system with an axial field-of-view of 22.1 cm and transaxial FOV of 81.4 (Rausch et al., 2015) and included a CT image for attenuation and scatter correction. The attenuation correction map was used to generate the material maps used in the PRC method by thresholding different linear attenuation coefficient ranges for 511 keV photons (below 0.08 cm^−1^ was considered lung material, between 0.08 cm^−1^ and 0.12 cm^−1^ as water and above 0.12 cm^−1^ the remaining area was treated as bone). PET raw data was recorded in list mode. The phantom scans using ^18^F and ^124^I were performed at the University Clinic in Essen, Germany. Scans with ^68^Ga were performed at the Royal North Shore Hospital in Sydney, NSW, Australia.

Emission scan time for the ^18^F and ^124^I phantoms was 60 min, while that for the ^68^Ga-filled phantom was 13 min. To allow an objective comparison of the OSEM and PRC image reconstructions, the reconstruction settings were selected to yield a comparable image noise level, ~10% for ^18^F and ^124^I images and ~ 15% for the ^68^Ga images as a clinically relevant parameter (Boellaard et al., 2016; Koopman et al., 2016). This was achieved for the standard OSEM algorithm with 2 iterations and 12 subsets and the developed PRC with 8 iterations and 12 subsets for ^68^Ga and ^124^I and 3 iterations and 12 subsets for ^18^F using both OSEM and PRC, except otherwise stated. A 400x400x109 image matrix was used, thus, resulting in a voxel size of 2.036 × 2.036 × 2.027 mm^3^. All standard corrections (attenuation, scatter including relative scatter scaling, randoms and normalization) were applied as implemented by the vendor. The reconstructions were done including the time-of-flight (TOF) information and no post-reconstruction filter was applied.

#### NEMA Image Quality Phantom

**Figure 3:**
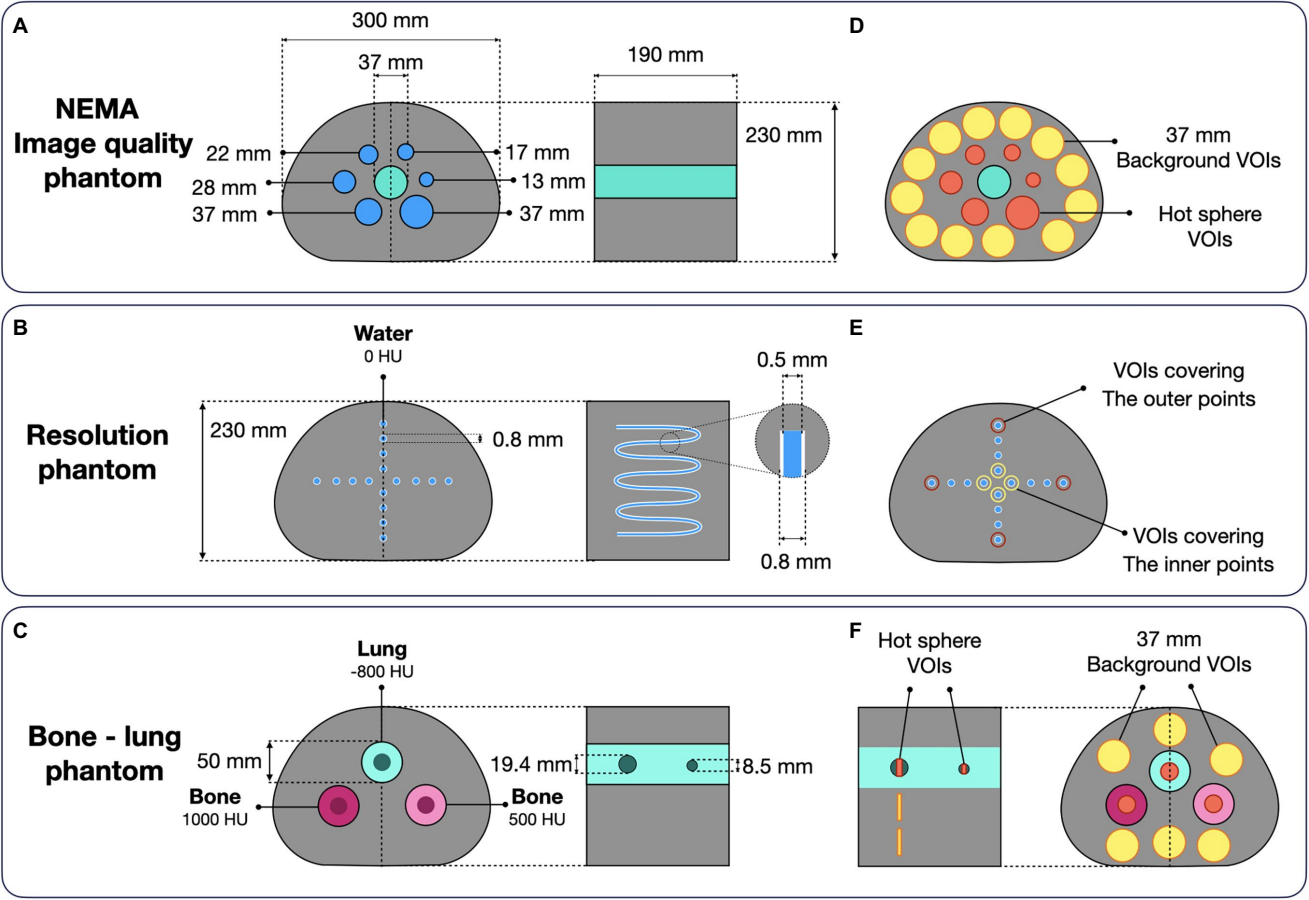
Experimental phantom designs: **(A)** NEMA image quality phantom (evaluated with ^18^F, ^124^I, and ^68^Ga); **(B)** Resolution phantom made of a 0.8-mm tube insert in the NEMA image quality housing (^18^F and ^124^I); **(C)** Bone–lung phantom with two spherical hot inserts placed in cold cylindrical tubes (^124^I). Corresponding ROI definitions for subsequent data analysis are shown in **(D–F)**.

NEMA IQ (Figure 3A) measurements were performed with three different radionuclides ^18^F, ^68^Ga, and ^124^I. The phantoms were filled with an activity concentration of 30 kBq/ml in the spheres and 6 kBq/ml in the background (activity ratio of 5:1) for the ^18^F and ^124^I measurements. For ^68^Ga, 24 kBq/ml was used for the spheres and 3 kBq/ml in the background (activity ratio of 8:1). Additional image reconstructions with iterations from 1 to 10 with 12 subsets were performed for the ^18^F and 1 to 30 iterations with 12 subsets for the ^68^Ga and ^124^I NEMA IQ phantoms to evaluate the convergence of the PRC in comparison to the standard OSEM. For the analysis, spherical volumes of interest (VOI) covering the hot spheres (diameter of 10, 13, 17, 22, 27, and 37 mm) and 12 background VOIs (diameter of 37 mm) were manually placed in the uniform background region (Figure 3D) using Amide 1.0.5 (AMIDE’s Medical Image Data Examiner).

The effect of PRC was analyzed for image noise:


(5) $$Noise=\frac{STD_{background}}{Mean_{background}}\times 100$$

where *STD_background_* was calculated as the mean of the standard deviation of the individual background VOIs divided by the mean values within the background VOIs.

Further, image contrast was defined as:


(6) $$Contrast=\frac{Mean_{signal}}{Mean_{background}}$$

where the mean value of the sphere VOI of the signal was calculated in the hot spheres divided by the mean values in the background VOIs similar as defined in the NEMA NU2 protocols.

When analyzing the convergence of the methods, the contrast-to-noise ratio (CNR) was calculated:


(7) $$CNR=\frac{Mean_{signal}-Mean_{background}}{STD_{background}}$$

In addition, the contrast recovery coefficient was assessed as:


(8) $$Recovery\;coefficient=\frac{Contrast}{Activity\;ratio}$$

where the contrast was defined as in Eq. 6 and the activity ratio was calculated as the ratio of the actually filled activity (measured with the dose calibrator) in the hot lesions to the actual activity in the background.

Finally, for the reconstructed images, which were selected for the direct comparison between the OSEM and PRC reconstruction the contrast recovery was calculated as:


(9) $$Contrast=\frac{\frac{Mean_{sphere}}{Mean_{background}}-1}{\frac{Activity_{sphere}}{Activity_{background}}-1}*100$$

#### Resolution Phantom

To assess the effect of PRC on spatial resolution, a phantom made of a tube structure built in the housing of the NEMA IQ phantom was used (Figure 3B). The tubes were made of polyethylene, with an inner diameter of 0.5 mm and a 0.15 mm wall thickness. The tube structure of the resolution phantom mimics 16-point sources in transverse direction. Phantom measurements were performed using ^18^F and ^124^I. The acquired data were reconstructed with OSEM and PRC with iterations from 1 to 10 with 12 subsets. The full-width at half maximum (FWHM) was calculated for the inner- and outer four-point sources in a central plane (Figure 3E). The images reconstructed with the predefined optimal reconstruction settings were compared.

#### Bone–Lung Phantom

The bone–lung phantom consists of a NEMA IQ housing with 3 cylindrical inserts with a diameter of 50 mm (Figure 3C). The inserts were filled with different materials mimicking lung and two bone types with attenuation coefficients (AC) expressed in Hounsfield units (HU) of −800 HU (lung), 500 HU (bone), and 1,000 HU (bone). In every cylinder, two fillable spherical inserts (*d* = 8.5 mm and 19.4 mm) were inserted. The phantom was filled with an activity concentration of 30 kBq/ml in all the spheres and 6 kBq/ml in the background region (activity ratio of 5:1). The experiments were conducted with ^18^F and ^124^I, and data evaluation was performed by extracting the activity from VOIs covering the hot spheres (8.9 and 19.4 mm) and 6 background VOIs (*d* = 37 mm; Figure 3F). The recovery coefficient (Eq. 8) of the hot spheres was calculated.

For verification purposes, the proposed PRC method was evaluated in comparison with two simplified PRC approaches. First, a uniform water PR kernel was used for the entire image (PRC-Unif), and second, a spatially invariant only tissue-dependent PR modeling (PRC-TD) only taking into account the different tissues but ignoring tissue borders was included in the image reconstruction. The implementation was the same as described in Eq. 4. And the recovery coefficients were reported for the 8.5 and 19.4 mm hot spheres.

## Results

### Simulating Positron Range Distributions

**Figure 4:**
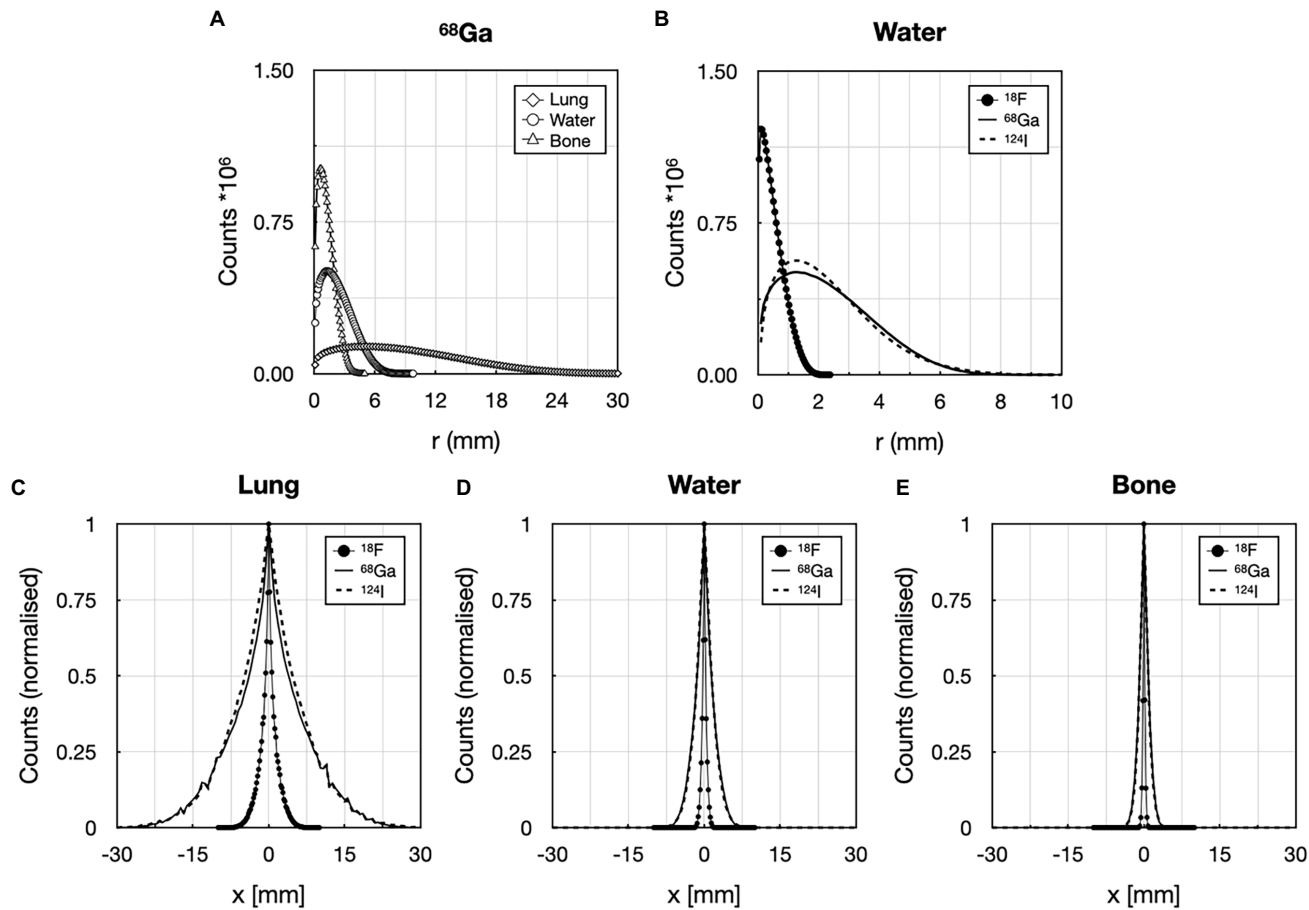
Comparison of the 3D positron ranges for: **(A)** ^68^Ga in lung, water, and bone material (20 million annihilation events), and **(B)** for ^18^F, ^68^Ga, and ^124^I (20 M annihilation events) in water. Normalized line profiles through the center of the 3D kernel in different materials: **(C)** lung, **(D)** water, and **(E)** bone for ^18^F, ^68^Ga, and ^124^I. Positron range blurring kernels took into account the maximum range of the given radionuclide in lung material.

The mean positron ranges in water calculated from the MC simulations were 0.50 mm (^18^F), 2.32 mm (^68^Ga), and 2.28 mm (^124^I). Figure 4A shows examples of the simulated PR for ^68^Ga. Figures 4C–E presents line profiles through the center slices along the *z*-axis in the *xy* plane of the 3D PR distributions for all isotopes and materials. Table 1 summarizes the mean PR in all simulated materials including a comparison of mean PR values presented in literature.

**Table 1:** Simulated mean PR for the ^18^F, ^68^Ga, and ^124^I in lung, water, and bone compared to the published values.

Radionuclide	Material	This work	Cal-González et al., 2013	Bertolli et al., 2016	Emond et al., 2019	Carter et al., 2020	Caribé et al., 2020
		GATE 9.0	Penelope	GATE	GATE 8.2	PHITS v3.02	GATE
		Mean [mm]	Mean [mm]				
^18^F	Lung	1.96	1.85	2.14	–	1.41	2.23
	Water	0.50	0.57	0.55	0.44	0.42	0.50
	Bone	0.22	0.32	–	–	0.22	0.34
^68^Ga	Lung	8.97	8.86	9.69	–	8.01	8.09
	Water	2.32	2.69	2.54	2.39	2.41	2.32
	Bone	1.18	1.44	–	–	1.26	1.33
^124^I	Lung	8.84	–	–	–	–	–
	Water	2.28	–	–	2.70	–	–
	Bone	1.16	–	–	–	–	–

*The main reason for the residual differences is due to the positron cross sections defined in the simulation frameworks*.

The kernel sizes were set to 11 × 11 × 11 pixels given the voxel size (2.036 × 2.036 × 2.027 mm^3^) used for the PET reconstructions and the maximum PR ranges in lung of ~10 mm for ^18^F, ~10 mm for ^68^Ga and ^124^I in water.

### Evaluation of the Spatially Variant PR Kernels

**Figure 5:**
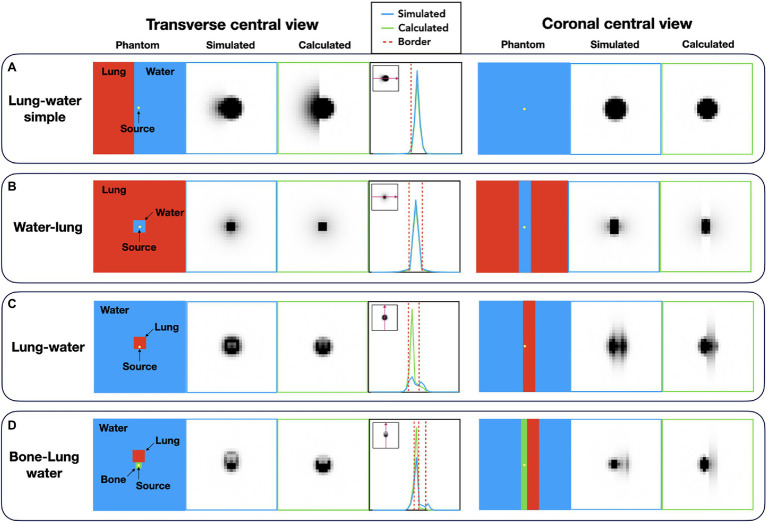
Comparison of the simulated and calculated spatially variant, tissue-dependent kernels. **(A)** Lung–water simple phantom; **(B)** water–lung phantom; **(C)** lung–water phantom; and **(D)** bone–lung–water phantom. The differences appear at the edges of the kernels when the positron traverses from a higher density medium to a lower density material.

The simulated and calculated tissue-dependent and spatially variant kernels were visually similar (Figure 5). However, local deviations were observed mainly at material borders, with the highest deviations seen when the positron travels from a lower density material to a higher density region. In some cases, the annihilation peak in the source center was lower for the simulated kernels compared to the calculated kernels (Figures 5C,D). These changes were also seen on the edges of the kernel (Figure 5A).

### Experimental Validation

#### NEMA IQ Phantom

**Figure 6:**
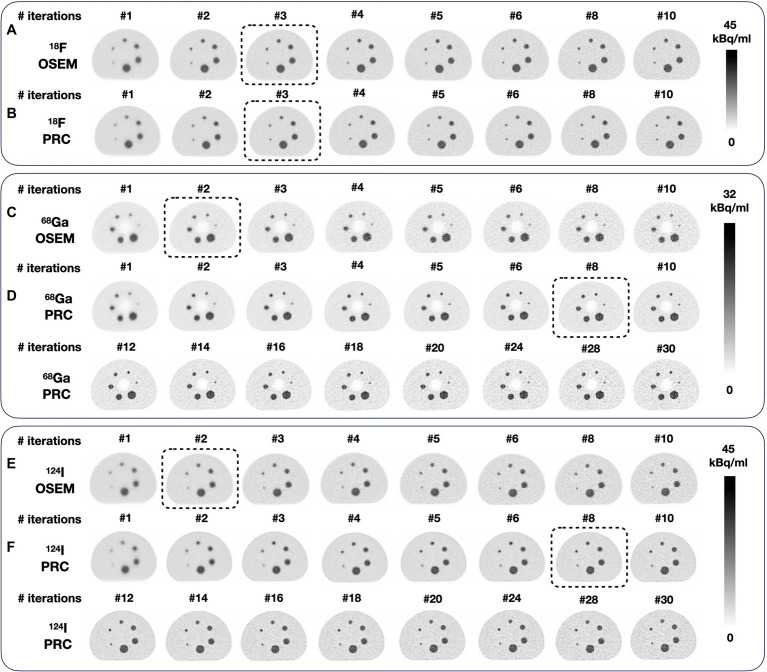
Reconstructions of the NEMA image quality phantom using different numbers of iterations: **(A)** ^18^F and OSEM; **(B)** ^18^F and PRC; **(C)** ^68^Ga and OSEM, and **(D)** ^68^Ga and PRC; **(E)** ^124^I and OSEM, and **(F)** ^124^I and PRC. Similar noise levels (~10%) were achieved for ^124^I and (~15%) for ^68^Ga with OSEM 2 iterations and PRC 8 iterations (both with 12 subsets). For the phantom filled with ^18^F, similar noise levels were achieved at 3 iterations (12 subsets) for both OSEM and PRC. The images selected for direct comparison are marked with dashed boxes.

**Figure 7:**
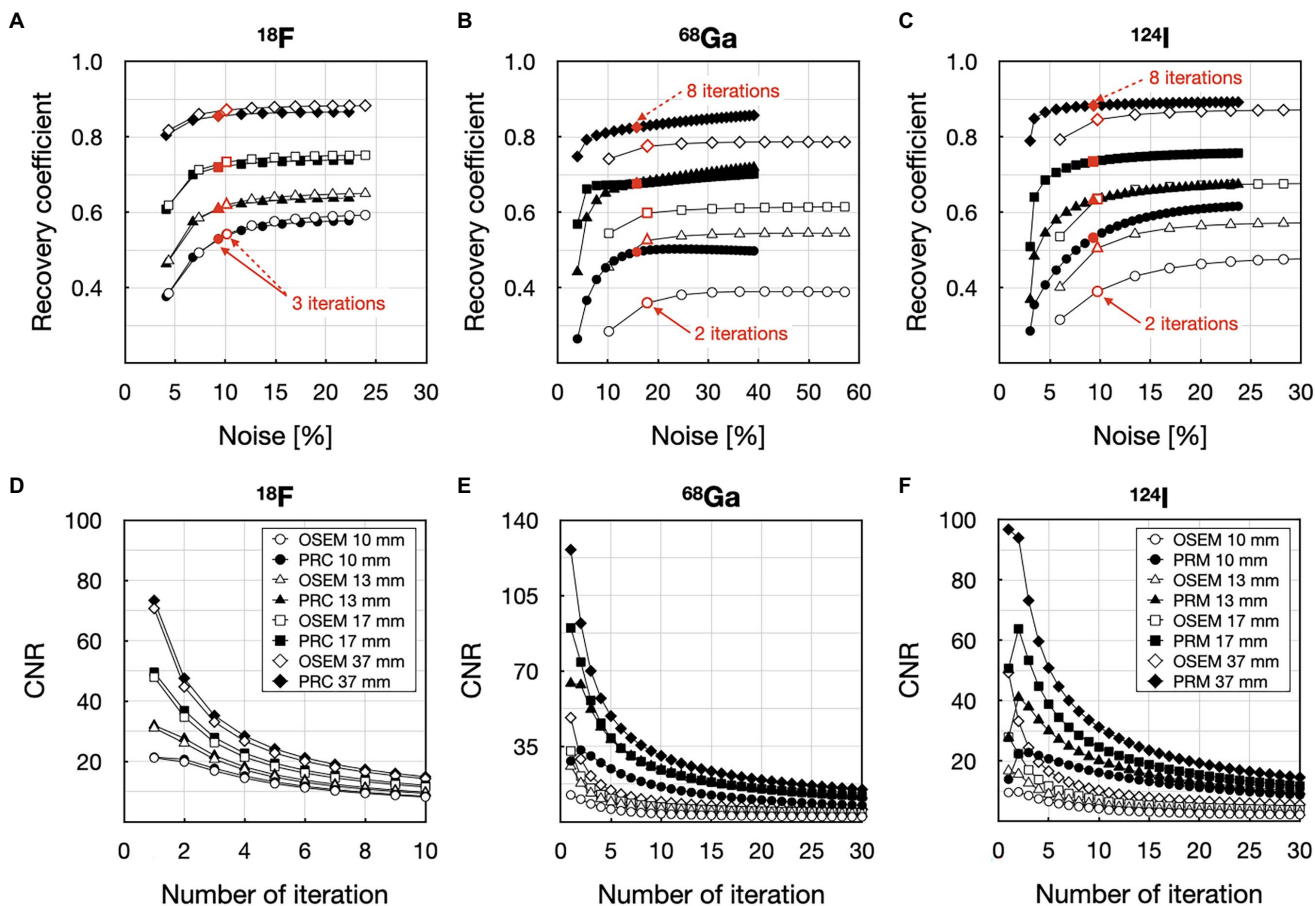
Contrast vs. noise analysis of the 10 mm, 13 mm, 17 mm and 37 mm spheres in the NEMA IQ phantom filled with ^18^F **(A,D)**, ^68^Ga **(B,E)**, and ^124^I **(C,F)**: **(A)** recovery coefficient vs. noise with ^18^F; **(B)** recovery coefficient vs. noise with ^68^Ga; **(C)** recovery coefficient vs. noise with ^124^I; **(D)** contrast-to-noise (CNR) ratio as a function of the number of iterations with ^18^F; **(E)** CNR ratio as a function of the number of iterations with ^68^Ga; **(F)** CNR ratio as a function of the number of iterations with ^124^I. Empty symbols and solid symbols represent the OSEM reconstructions without and with and PRC, respectively.

**Figure 8:**
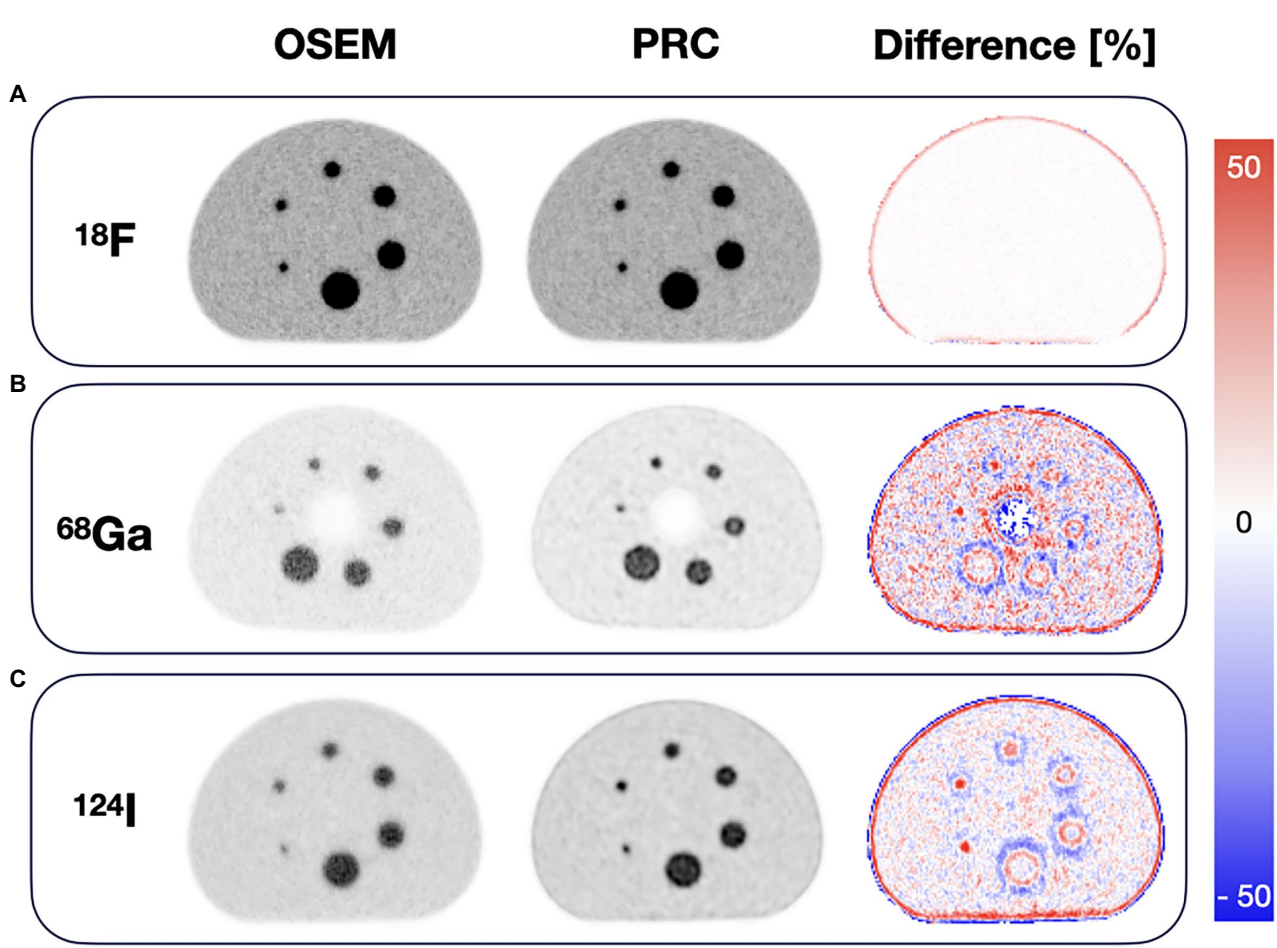
Comparison of the images of the NEMA image quality phantom with OSEM and PRC for **(A)** ^18^F, **(B)** ^68^Ga, and **(C)** ^124^I acquisitions. ^18^F images (both OSEM and PRC) were reconstructed with 3 iterations and 12 subsets, while ^68^Ga and ^124^I images with 2 iterations and 12 subsets for OSEM and 8 iterations as 12 subsets for PRC. The largest differences were seen at the boundaries between areas of different activity concentrations.

The visual comparison of the NEMA IQ phantom filled with ^18^F, ^68^Ga, and ^124^I reconstructed with different number of iterations (1–10 for ^18^F) and (1–30 for ^68^Ga and ^124^I) with 12 subsets using both the standard OSEM and PRC reconstructions is depicted on Figure 6. The evaluation of the convergence revealed an almost negligible effect of PRC for ^18^F acquisitions with similar recovery coefficients and noise levels at all iterations (Figure 7). PRC applied to ^124^I data showed a slower convergence than OSEM, however, with higher recovery coefficients and lower noise levels than those obtained with OSEM at higher iterations (Figure 7). At the same number of iterations, the CNR was improved for all the spheres with PRC compared to the standard OSEM for both ^18^F and ^124^I (Figures 7A,C). Nevertheless, Gibbs artifacts were present in the PRC images as seen most prominently in Figure 8 at the outer border of the phantom for ^68^Ga and ^124^I PRC reconstructions.

To compare the NEMA IQ measurements with ^18^F, ^68^Ga and ^124^I reconstructed with OSEM and PRC, the reconstruction settings were selected in order to produce similar noise levels of ~10% (Figure 6). For ^18^F, this was achieved with 3 iterations and 12 subsets for both OSEM and PRC reconstructions. In case of ^124^I, noise levels of 10% were found when using 2 iterations and 12 subsets for the OSEM reconstructions and 8 iterations as 12 subsets for PRC. These settings were used in the following also for the ^68^Ga acquisition due to the similar PR of ^68^Ga and ^124^I.

Using PRC, the improvements in recovery coefficients ranged from 6 to 33% and 4 to 24% for ^68^Ga and ^124^I, respectively. Maximal recovery coefficient was found for the 10- and 13-mm spheres, respectively. In the case of ^18^F, the contrast changes were negligible and are slightly reduced (maximum with 2% for the 10 mm sphere); on the other hand, the image noise was reduced as well. A summary of all contrast recovery and recovery coefficients for all spheres and isotopes can be found in Table 2. In general, the influence of PRC was seen at borders between areas of different activity concentrations, for example, at the edges of the phantom, and was more pronounced for the radionuclides with the longer PR (Figure 8).

**Table 2:** Calculated recovery coefficient and contrast recovery and background noise for the images of NEMA IQ phantom for different image reconstruction and radionuclide combinations.

Sphere size	Radionuclide	Recovery coefficient			Contrast recovery [%]	
		OSEM	PRC	Relative difference [%]	OSEM	PRC
10 mm	^18^F	0.54	0.53	−2.34	42.8	41.2
	^68^Ga	0.37	0.49	32.51	28.4	42.2
	^124^I	0.39	0.53	36.52	23.8	41.6
13 mm	^18^F	0.62	0.61	−1.8	52.5	51.1
	^68^Ga	0.56	0.68	21.5	49.3	63.0
	^124^I	0.50	0.63	25.0	38.1	53.9
17 mm	^18^F	0.73	0.72	−1.8	66.6	64.9
	^68^Ga	0.61	0.68	10.1	55.8	62.9
	^124^I	0.64	0.73	15.4	54.5	66.8
22 mm	^18^F	0.82	0.80	−1.7	77.2	75.5
	^68^Ga	0.67	0.73	7.7	62.7	68.6
	^124^I	0.75	0.81	8.2	68.8	76.5
28 mm	^18^F	0.84	0.81	−2.9	79.6	76.6
	^68^Ga	0.72	0.79	8.7	68.5	75.8
	^124^I	0.77	0.83	7.8	71.5	79.1
37 mm	^18^F	0.87	0.85	−1.8	83.9	81.9
	^68^Ga	0.78	0.82	5.5	75.1	80.0
	^124^I	0.85	0.88	4.4	80.7	85.3
Background noise [%]						
	^18^F	10.1	9.30	−8.3		
	^68^Ga	17.7	15.7	−11.0		
	^124^I	9.7	9.35	−3.8		

*^18^F images (both OSEM and PRC) were reconstructed with 3 iterations and 12 subsets, while ^68^Ga and ^124^I images with 2 iterations and 12 subsets for OSEM and 8 iterations as 12 subsets for PRC*.

#### Spatial Resolution Phantom

**Figure 9:**
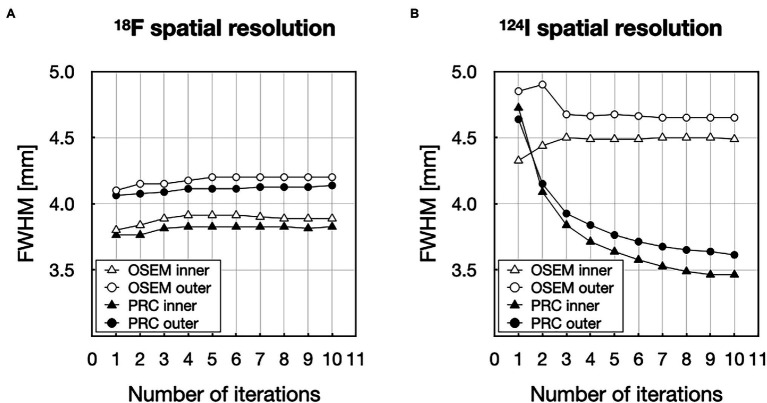
Evaluation of the spatial resolution represented as the number of iterations (1–10) vs. full-width at half-maxim (FWHM) for **(A)** ^18^F and **(B)** ^124^I.

**Figure 10:**
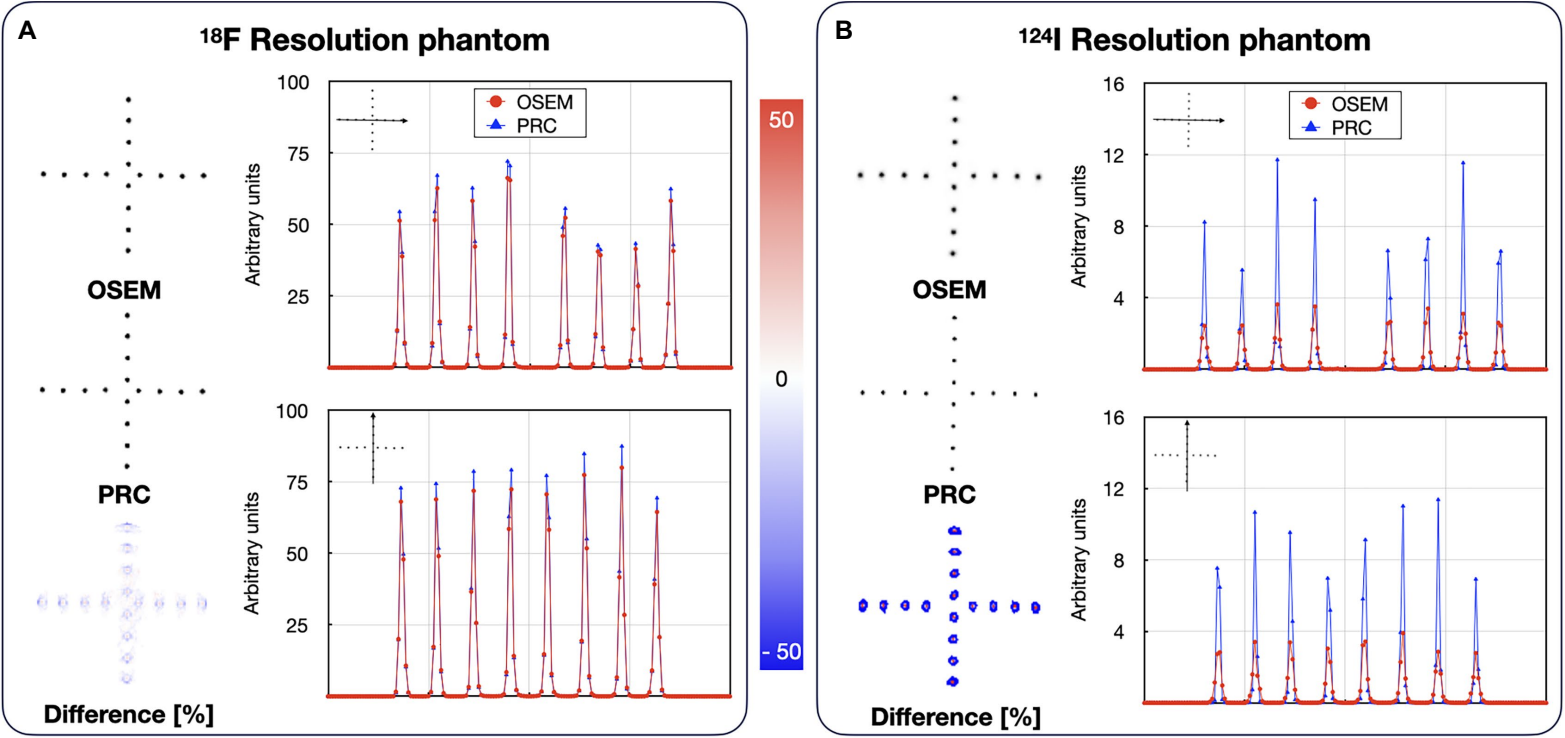
Reconstructed images of the resolution phantoms filled with: **(A)** ^18^F and **(B)** ^124^I. Both cases are reconstructed with OSEM and PRC. ^18^F images (OSEM and PRC) were reconstructed with 3 iterations and 12 subsets, while ^124^I images with 2 iterations and 12 subsets for OSEM and 8 iterations as 12 subsets for PRC. The line profiles are calculated through vertical and horizontal spheres indicated by the arrows. The effect of PRC is notably higher in the ^124^I.

PRC did only marginally influence the spatial resolution for ^18^F independent of the number of iterations (Figure 9A), similar to the recovery coefficient findings in the NEMA IQ experiments. However, spatial resolution improved remarkably for ^124^I, depending on the number of iterations (Figure 9B). More specifically, when reconstructing the images following the previously defined settings, for ^18^F, the spatial resolution improvements were below 2% with a mean FWHM of 3.9 ± 0.2 mm and 3.8 ± 0.2 mm for the inner and 4.2 ± 0.2 mm and 4.1 ± 0.1 mm for the outer point source for OSEM and PRC, respectively. For ^124^I, improvements in FWHM up to 26% were observed with FWHM changes from 4.4 ± 0.3 mm to 3.5 ± 0.2 mm for the inner- and 4.9 ± 0.5 mm to 3.7 ± 0.4 mm for the outer point sources between OSEM and PRC, respectively. The visual differences between the reconstructed images are shown in Figure 10. The improvements were clearly visible on the vertical and horizontal line profiles through the point sources of ^124^I with minor changes for ^18^F.

#### Bone–Lung Phantom

**Figure 11:**
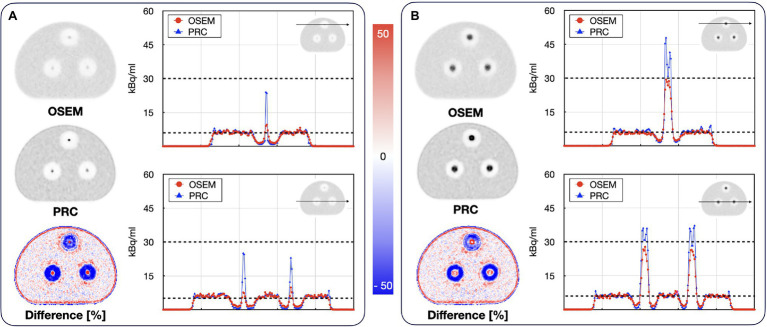
Reconstructed images of the Bone–lung phantom filled with ^124^I **(A)** slice through the 8.5 mm spheres; **(B)** slice through the 19.4 mm spheres. The line profiles show the activity concentrations along the depicted lines through the lung and bone mimicking cylindrical inserts. The black dashed lines indicate the actual activity levels in the background and the hot spheres. The effect of PRC is notably higher in low-density regions (lung) for the large spheres and more pronounced in the bone medium for the smaller spheres.

Recovery coefficients of all spherical inserts did improve with PRC applied to the ^124^I experiments (Table 3). This was similar to the effects seen for the NEMA IQ phantom. Here, recovery coefficient of the 8.5 mm spherical insert increased by 191, 89, and 115% for lung, bone (500 HU), and bone (1,000 HU), respectively. For the 19.4 mm insert, improvements of 42, 18, and 16%, respectively, were calculated. Changes in activity distribution appear most prominently on the phantom edges and around the hot inserts (Figure 11).

**Table 3:** Recovery coefficient for the hot small spheres (*d* = 8.5 mm) and hot large spheres (19.4 mm) of the Bone–lung phantom reconstructed with OSEM (2 iterations and 12 subsets) and PRC-Unif, PRC-TD, and PRC (8 iterations and 12 subsets).

Material	Recovery coefficient					
	8.5 mm			19.4 mm		
	Lung (−800 HU)	Bone (500 HU)	Bone (1,000 HU)	Lung (−800 HU)	Bone (500 HU)	Bone (1,000 HU)
OSEM	0.20	0.18	0.17	0.60	0.59	0.59
PRC-Unif	0.39	0.38	0.33	0.70	0.74	0.75
PRC-TD	0.32	0.42	0.34	0.66	0.75	0.76
PRC	0.57	0.33	0.36	0.85	0.70	0.68
Relative difference OSEM-PRC [%]	191	89	115	42	18	16

**Figure 12:**
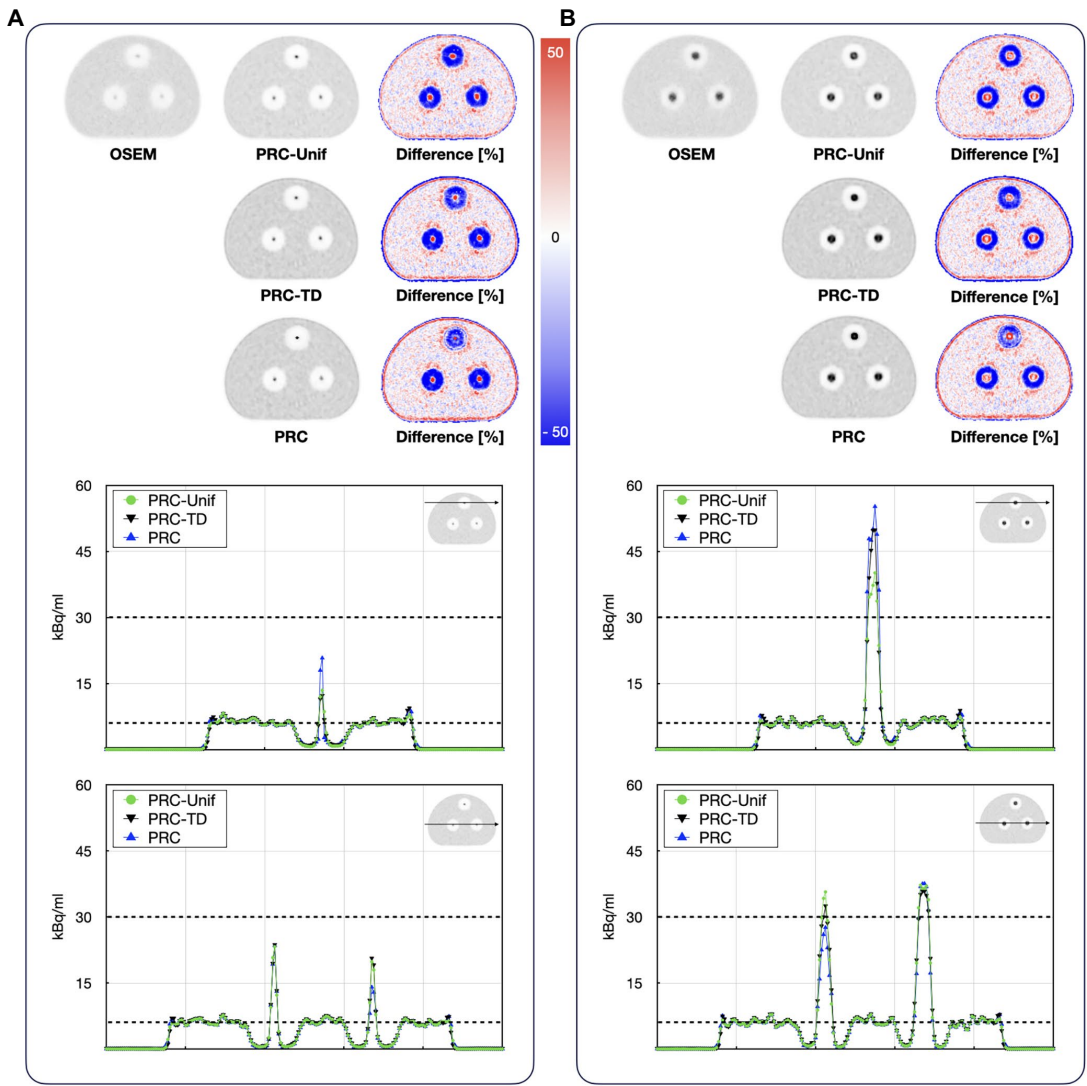
Reconstructed images of the bone–lung phantom filled with ^124^I using PRC-unif, PRC-TD, and PRC: axial images of the **(A)** 8.5 mm spheres and **(B)** 19.4 mm spheres. The black dashed lines indicated the actual activity levels in the background and the hot spheres.

The comparison of the different PR modeling methods is shown in Figure 12. For the hot spheres embedded in bone material, all PRC methods yielded similar recovery coefficients between 32 to 42% and 68 to 76% for the 8.5 and 19.4 mm spheres, respectively. For the spheres embedded in lung material, PRC deviated from the PRC-Unif and PRC-TD-based reconstructions resulting in a higher recovery coefficient for both in the 8.5 mm and 19.4 mm sphere (Table 3) whereby the contrast in the 19.4 mm sphere was overestimated.

## Discussion

In this study, we successfully implemented a versatile positron range correction into clinical reconstruction software. The PR effects were simulated using the GATE MC simulation framework for relevant radionuclides and tissue types. We presented a new method for calculating the tissue-dependent and spatially variant positron range kernels implemented as a PSF in the iterative reconstruction process. Using this approach, substantial improvements in recovery coefficient for ^68^Ga and ^124^I acquisitions were demonstrated in experimental phantom settings.

The simulated positron range values were compared to the literature when using various simulation software; they were mostly in good agreement with published values (Table 1). Deviations can be attributed to the use of different physics definitions within the MC simulation frameworks (containing the positron cross sections, step size) and the definition of the materials (composition and mass density), as well as the radioactive source definitions. The largest deviation was −36% for ^18^F in bone material when comparing the PR to the published values by Caribé et al. (2020). For PRs in water, the same values were calculated for both ^18^F and ^68^Ga. Following the PR ranges in lung material, the PR kernel sizes were set to 11 × 11 × 11 voxels for ^18^F. In case for radionuclides with higher PR kernel, sizes were set to the maximum PR in water medium: 11x11x11 for ^68^Ga and ^124^I due to their similar PRs (Figure 4).

We estimated PR kernels from the material map and the uniform kernels derived from the MC simulations, which is in contrast to previous studies, such as Cal-González et al. (2015), who estimated the spatially variant tissue-dependent PR kernels from scaling kernels by their electron density, by calculating the mean density across the emission and annihilation voxels. The resulting spatially variant and tissue-dependent kernels were in good agreement with the simulated kernels (Table 1). However, the calculated kernels do not consider the energy loss of positrons in the material where they have been emitted. Thus, when crossing the border between the materials, the PR in the second material corresponds to the energy of the positron in the uniform region. This causes an overestimation of voxel values in the kernels at material borders when the positron was emitted in a higher density material (bone or water) and entering a lower density material (lung; Figure 5). Nonetheless, these inaccuracies appear acceptable given the improvement in calculation speed while keeping the kernels sized mapped to the maximum PR in water. Of note, the individual positron range kernels were calculated for every voxel of the image through the iterative image reconstruction process. Due to the large size of the total number of kernels (more than 17 M for the mCT PET/CT system given the large image size of 400 × 400 × 109 voxels), these cannot be stored easily, and, therefore, have to be recalculated for each iteration.

In contrast to previous studies where the PR blurring was applied only before the forward projection (Cal-González et al., 2011; Bertolli et al., 2016; Cal-Gonzalez et al., 2018b), our PR blurring kernels were implemented in both forward and back projection steps within the iterative reconstruction algorithm. Implementing the PR blurring only in the forward projection step appears to be an acceptable solution for small animal PET imaging (Cal-González et al., 2011). However, this approach may no longer be acceptable for PET imaging of humans, since—from a mathematical point of view—the kernels must be applied in both steps, as the back projector is the transpose of the forward projection operator (Eq. 4). Nevertheless, the full implementation of PR substantially increases the image reconstruction time.

After incorporating the PR blurring kernels in the iterative process, the suitable image reconstruction settings were defined by analyzing the convergence or the reconstruction for ^18^F and ^124^I (Figure 7). To match the noise properties, 8 iterations and 12 subsets were required with PRC compared to 2 iterations and 12 subsets for the standard OSEM reconstruction in case of ^68^Ga and ^124^I (Figure 6). For ^18^F, this was achieved with 3 iterations (12 subsets) for both OSEM and PRC. This finding suggests that the convergence of PRC depends on the positron range kernel, and thus, the ideal reconstruction settings must be tailored to the used PET radionuclide and respective positron range. The slower convergence can be balanced if the same recovery coefficient is matched between the OSEM and PRC reconstructions. In this case, 8 iterations (12 subsets) are needed for OSEM and 4 iterations (12 subsets) for PRC for the ^124^I images (Figure 7C; 10 mm sphere) for similar recovery coefficient and lower image noise. As shown in previous studies if the PR correction is applied only before the forward projection the convergence is faster, however the image noise is increased. In the presented method, this is balanced by adding the PR blurring to the back projection, which acts also similar as a regularization, suppressing the image noise.

The behavior of PRC was comparable to the one of PSF corrections for which similar changes in the convergence were reported before (Panin et al., 2006). This is reasonable, as the implementation of the positron range blurring kernel in the OSEM algorithm in both forward and back projection steps was done similarly for PSF corrections (Panin et al., 2006).

In general, the PRC was able to substantially reduce the PR effect, and, thus, increase image contrast and spatial resolution. In the NEMA IQ phantom, this was most prominently seen for the smaller spheres with increases in contrast by up to 33% for the 10-mm sphere when using ^68^Ga (Figure 8). In line with these results, spatial resolution improvements of up to 26% were observed for ^124^I within the image resolution phantom (Figures 9, 10).

However, PRC is prone to edge artifacts, also known as Gibbs artifacts (Nuyts, 2014). Such artifacts were noticed in this study at the edges of the larger spheres in the NEMA IQ phantom (Figure 8) and at the edges of the evaluated bone–lung phantom (Figure 11). This effect is in particular evident in the 22 mm spheres similar as already reported for PSF reconstructions by Knäusl et al., 2013. Gibbs artifacts are known from PSF reconstructions, and, thus, are not unexpected in the PRC, as the presented method acts as an additional PSF within the image reconstruction (Nuyts, 2014). These artifacts are expected to originate from slightly inaccurate positron range kernels, and, thus, a mismatch with the actual PSF of the PET system (Tong et al., 2011; Rahmim et al., 2013). The Gibbs artifacts could also explain the improved image resolution after PRC for ^124^I in the resolution phantom when compared to the ^18^F experiments (Figure 9). However, the magnitude of these artifacts may be reduced by using application-specific, post-filtering of the images (Knäusl et al., 2013), or by reducing the sizes of the PR kernels from the applied 11 × 11 × 11 to 5 × 5 × 5 pixels. Furthermore, these effects are known to be visible in phantoms where sharp borders between different activity areas exist, but they may not be noticeable in-patient imaging.

The comparison of the PRC with more simple methods only assuming a uniform PR throughout all materials (PRC-Unif) or using a tissue-dependent PR without taking into account tissue borders (PRC-TD) revealed a similar performance of all methods within lesions embedded in bone material (Table 3) with contrasts similar to does obtained with ^18^F (Table 2) when taking into account average differences of 5% points between repeated assessments of contrasts in NEMA IQ studies (Rausch et al., 2015). This is reasonable given the similarity of the PR kernels for water (mean PR for ^124^I = 2.3 mm) and bone (mean PR for ^124^I = 1.2 mm) when using an isotropic voxel size of 2 mm.

When applying PRC higher recovery coefficient was calculated in the 8.5 mm sphere within lung medium, outperforming PRC-Unif and PRC-TD (Table 3). The PRC method is expected to more accurately model the actual PR distribution at tissue borders than the simple methods tested. However, the recovery coefficient was slightly overestimated in the 19.4 mm sphere within lung medium. We hypothesize that this behavior is caused by a combination of three effects: The spherical inserts have a wall thickness of ~1 mm. Within the bone material, the impact on PRC is expected to be negligible as the density of the plastic material is in between the one of bone and water. However, in the lung insert the plastic walls [made of Polymethyl methacrylate (PMMA)] may have a non-negligible influence on PRC as they act as a comparatively high-density border between water and lung. This would also partly explain the similar contrast of the spheres in the different materials for OSEM reconstructions. A second effect, which may contribute to the findings is the scatter correction. The scatter simulation including the relative scatter scaling used to account for scatter from out of the field-of-view and also compensating for spurious coincidences inherent to the ^124^I decay (Conti and Eriksson, 2016) may not be able to correct all erroneous counts in cold areas such as the lung material. As the PRC method accounts for the longer PR in lung tissue for voxels within the hot sphere located near the tissue borders, erroneous count within the lung material can have a strong effect on the resulting PR corrected image. This effect is not expected to be prominent when using the simple methods as for all sphere voxels a water kernel is used. Similar is true also for PRC in the bone material as the PR range is low at the material borders. Thus, erroneous counts in the cold material are not transferred into the hot lesion. Finally, the strong overshooting of the contrast of the 19.4 mm sphere within the lung insert seems comparable to known effects caused by Gibbs artifacts in PSF reconstructions, where similar over corrections for spheres with diameters of 17 mm have been reported in unfiltered images (Knäusl et al., 2013) at high numbers of iterations.

### Limitations

The multiple core implementation of the kernel calculation and application helped reduce the processing time. The image reconstruction including the application of the PR blurring on the actual image and the back-projected image takes ~18 min/subset. Thus, using 6 iterations and 12 subsets, current implementation of PRC requires up to 22 h of reconstruction time per data set. In our case, this reconstruction speed was achieved when splitting the calculation task to 40 cores on a PC equipped with two Intel Xeon CPUs @2.6GHz. While this time seems reasonable for research purposes, substantial reductions of reconstruction time are required prior to adopting this PRC approach in clinical routine, especially for multiple bed position acquisitions. At this stage, the PRC reconstruction is done using a Matlab implementation accessing modified vendor and custom software parts. Furthermore, the PR correction is performed in image space by creating a voxel-specific PR kernel for every voxel in the reconstructed image. We believe that the reconstruction time can be significantly reduced by an optimized implementation, such as a comprehensive coding of all calculation steps in C++ and through parallelization of the kernel calculations using graphical processors (GPUs).

The local changes in tissue density were derived from the attenuation correction map. The AC maps have the same voxel size as the reconstructed PET images. Therefore, structures smaller than this voxel size cannot be corrected. This includes phantom parts, such as the tube housing for the resolution phantom, with a wall thickness of ~0.3 mm (spatial resolution phantom, Figure 3C); these parts are not visible, and, therefore, were treated as water medium in this implementation. This effect can lead to an overestimation of the positron ranges in these regions.

## Conclusion

The proposed method for positron range correction delivers a viable, spatially variant, and tissue-dependent correction method, which can be implemented for any clinical PET/CT system. Prior to clinical adoption, the kernel calculation must be optimized for efficiency, since computation time can be a serious limitation. Thanks to the resulting improvements in recovery coefficients and noise level reductions, this PRC implementation provides a promising correction for PET imaging of ^124^I- and ^68^Ga-labeled tracers, as demonstrated by a set of reference phantom studies.

## Data Availability Statement

The datasets generated and/or analysed during the current study are available from the corresponding author on reasonable request.

## Author Contributions

HK implemented the positron correction method, did the image reconstructions, the analysis, and evaluation of the data, and prepared the manuscript. TB and IR designed, guided, and supervised the project. JC-G provided the help with the simulations and gave expertise on the positron range effects. VP provided expertise in the implementation of the PRC into image reconstruction. WJ and PK performed the phantom measurements at different sites. AB helped with the Monte Carlo simulations. LP optimized the implementation of the PRC method. DB provided the projectors used for image reconstructions. JC and MC provided feedback on the development and evaluation of the results. HK, TB, VP, WJ, JC-G, AB, LP, PK, DB, JC, MC, and IR provided critical feedback and helped shape the research, analysis, and manuscript. All authors contributed to the article and approved the submitted version.

## Funding

The financial support of the Austrian FWF Project I3451-N32 is gratefully acknowledged. The authors acknowledge the support of Siemens Medical Solutions USA, Inc. (Knoxville, TN, United States).

## Conflict of Interest

VP, DB, JC, and MC are employees of Siemens Medical Solutions USA, Inc. (Knoxville, TN, United States) and report no conflict of interest with this study.

The remaining authors declare that the research was conducted in the absence of any commercial or financial relationships that could be construed as a potential conflict of interest.

## Publisher’s Note

All claims expressed in this article are solely those of the authors and do not necessarily represent those of their affiliated organizations, or those of the publisher, the editors and the reviewers. Any product that may be evaluated in this article, or claim that may be made by its manufacturer, is not guaranteed or endorsed by the publisher.
